# Endovascular treatment of an obstructive membrane between inferior vena cava and right atrium in an unrecognized Budd-Chiari syndrome

**DOI:** 10.1186/s42155-020-00168-5

**Published:** 2020-10-13

**Authors:** Maja Strozzi, Kristina Maric Besic, Knezevic Stromar Ivana, Anić Darko

**Affiliations:** 1 University Clinic for Cardiovascular Diseases, Clinical Hospital Center Zagreb, University of Zagreb School of Medicine, Zagreb, Croatia; 2 Department for Adult Congenital Heart Disease, University Clinic for Cardiovascular Diseases, Clinical Hospital Center Zagreb, Kispaticeva 12, 10000 Zagreb, Croatia; 3 Department of Gastroenterology, University Clinic for Internal Medicine, University Hospital Center, University of Zagreb School of Medicine, Zagreb, Croatia; 4 University Clinic for Heart Surgery, Clinical Hospital Center Zagreb, University of Zagreb School of Medicine, Zagreb, Croatia

**Keywords:** Inferior vena cava membrane, Intravenous percutaneous intervention, Budd-Chiari syndrome, Balloon angioplasty

## Abstract

**Background:**

Budd-Chiari syndrome is defined as a hepatic venous outflow track obstruction of various etiology, which appears at different levels. The inferior vena cava outflow membrane is an unusual, but a potentially treatable cause. The percutaneous treatment has emerged as a very promising management mode for such patients. Follow-up results are favorable for balloon angioplasty and/or stenting, with minimal re-stenosis rates.

**Case presentation:**

We report a case of a young woman, earlier operated on congenital heart defect and with previous pulmonary embolic incident after childbirth, with no evidence of thrombophilia. She was admitted to our institution for a suspected right atrial tumor. After the diagnosis of Budd-Chiari syndrome caused by membranous inferior vena cava obstruction, a percutaneous treatment of a thick membrane was successfully performed, using an unusual technique.

**Conclusion:**

Balloon angioplasty should be considered in cases of membranous obstruction of vena cava, where a focal obstruction is causing the symptoms. In our patient, the anatomy was not suitable for stenting, and balloon dilatation was successful just after the membrane was pulled apart with a big balloon in a “Rashkind-like” procedure.

## Background

Membranous obstruction of inferior vena cava is one of the causes of Budd-Chiari Syndrome (BCS). These membranes are thought to be a congenital abnormality, but they are more frequently acquired (Sevenet et al. 1989). Inferior Vena Cava (IVC) obstruction is more common in Asian male population, and in western countries BCS is found mostly in women and its predominant cause is hepatic vein obstruction (Plessier and Valla 2008). This syndrome encompasses various hepatic venous outflow blocks, one of which is membranous obstruction of the IVC. In contrast to hepatic vein thrombosis, formation of a membrane may be an outcome of recurrent thrombosis (Okuda 2001). BCS has been shown by the literature to be associated with pro-thrombotic states (Janssen et al. 2000).

Diagnosis can be established by clinical examination, Doppler ultrasound, computed tomography (CT), magnetic resonance imaging (MRI), and venography.

Obstructive membrane was predominantly treated through surgery but percutaneous transluminal balloon angioplasty is an alternative and effective form of treatment (Xu et al. 2004; Han et al. 2013). Balloon dilatation, with or without stenting of hepatic veins and/or IVC has been reported earlier by various authors (Srinivas et al. 2012). Percutaneous transluminal angioplasty for a complete membranous obstruction of the suprahepatic inferior vena cava is safe and effective, and the long-term results are excellent (Kucukay et al. 2016).

## Case presentation

We present a 45 years old woman with known congenital heart defect, who was transferred to our hospital with right atrial tumour suspicion and an unrecognized BCS.

A surgical closure of an atrial septal defect (ASD) was performed when she was 8 years old. After childbirth in 2008, she was treated with heparin for pulmonary embolism, followed by a short period of warfarin therapy (a few months). An echocardiographic examination (ECHO) was performed in 2009, after the patient complained of a certain effort intolerance, and a discrete leg edema. A recanalization of ASD was excluded, and the finding was described as within normal limits.

**Fig. 1:**
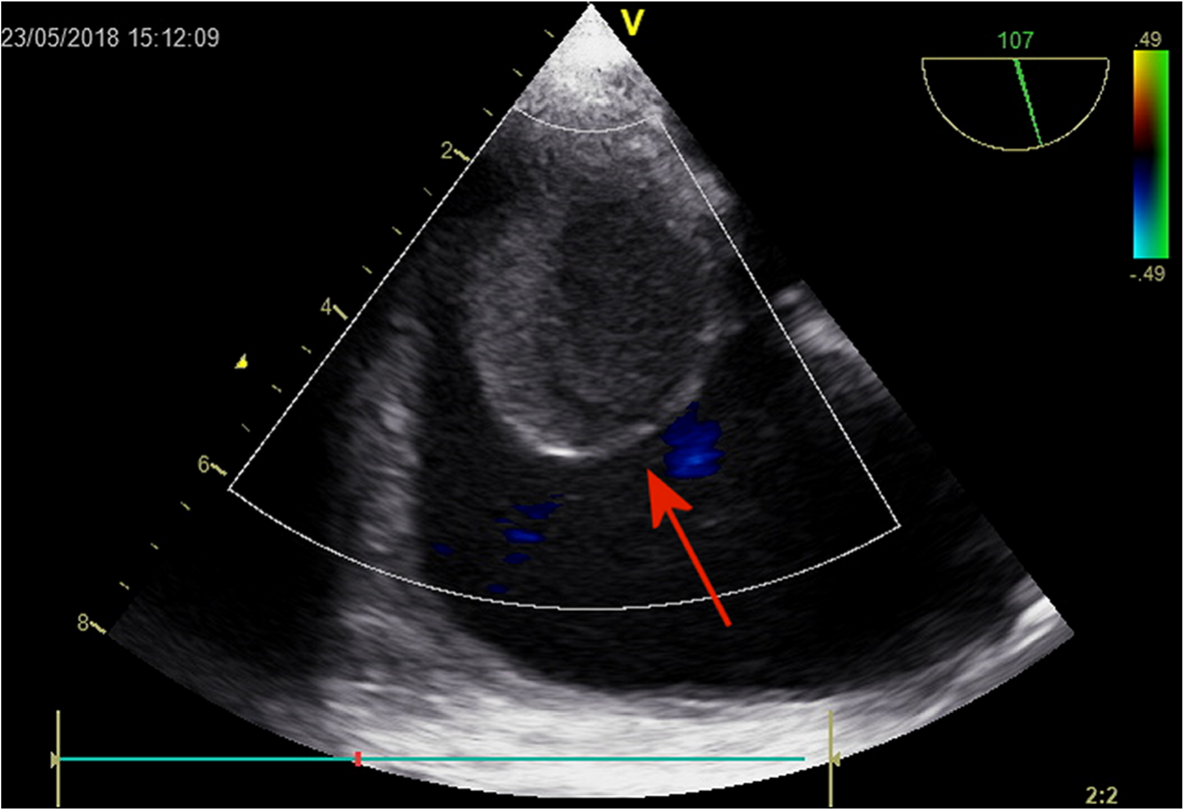
ECHO The first echo performed in a local hospital, indicated a possible tumour in the right atrium (arrow), what was the reason for referring the patient to our institution for further diagnostics and treatment

During the preceding 6 months, she had some serious clinical symptoms: a more severe dyspnea, cough, effort intolerance, ascites and leg swelling. She was admitted to a county hospital. The differential diagnosis was focused on heart failure, lung disease, and occult neoplasm. ECHO revealed a tumorous process in the right atrium 2.7 × 1.7 cm attached to the atrial septum (Fig. 1). She was transferred to our institution for further investigation and treatment.

**Fig 2:**
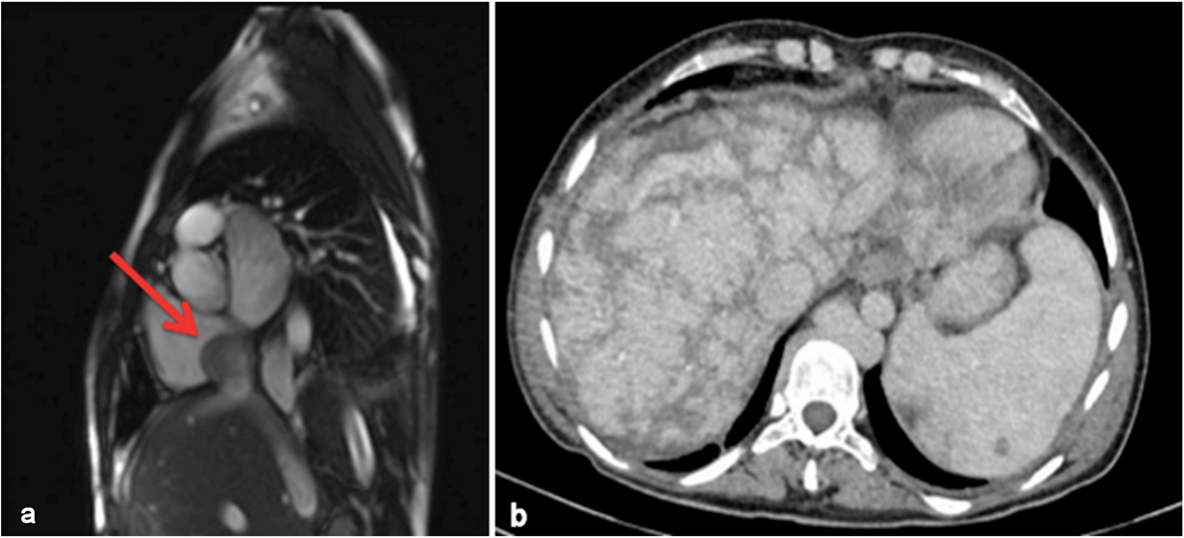
NMR: **a** IVC dilated (3,5–4,1 cm) separated from RA with a 3 mm thick membrane (arrow) obstructing the flow, **b** dilated hepatic veins

At admission, the patient’s clinical examination revealed a markedly distended abdomen with prominent superficial veins, bilateral pedal edema and hepato-splenomegaly. There was no jaundice. Clinical presentation was highly suspicious for BCS. ECHO (transthoracic and trans-esophageal), followed by MRI, revealed a membrane, dividing IVC inflow and right atrium, without communication (Fig. 2).

**Fig. 3:**
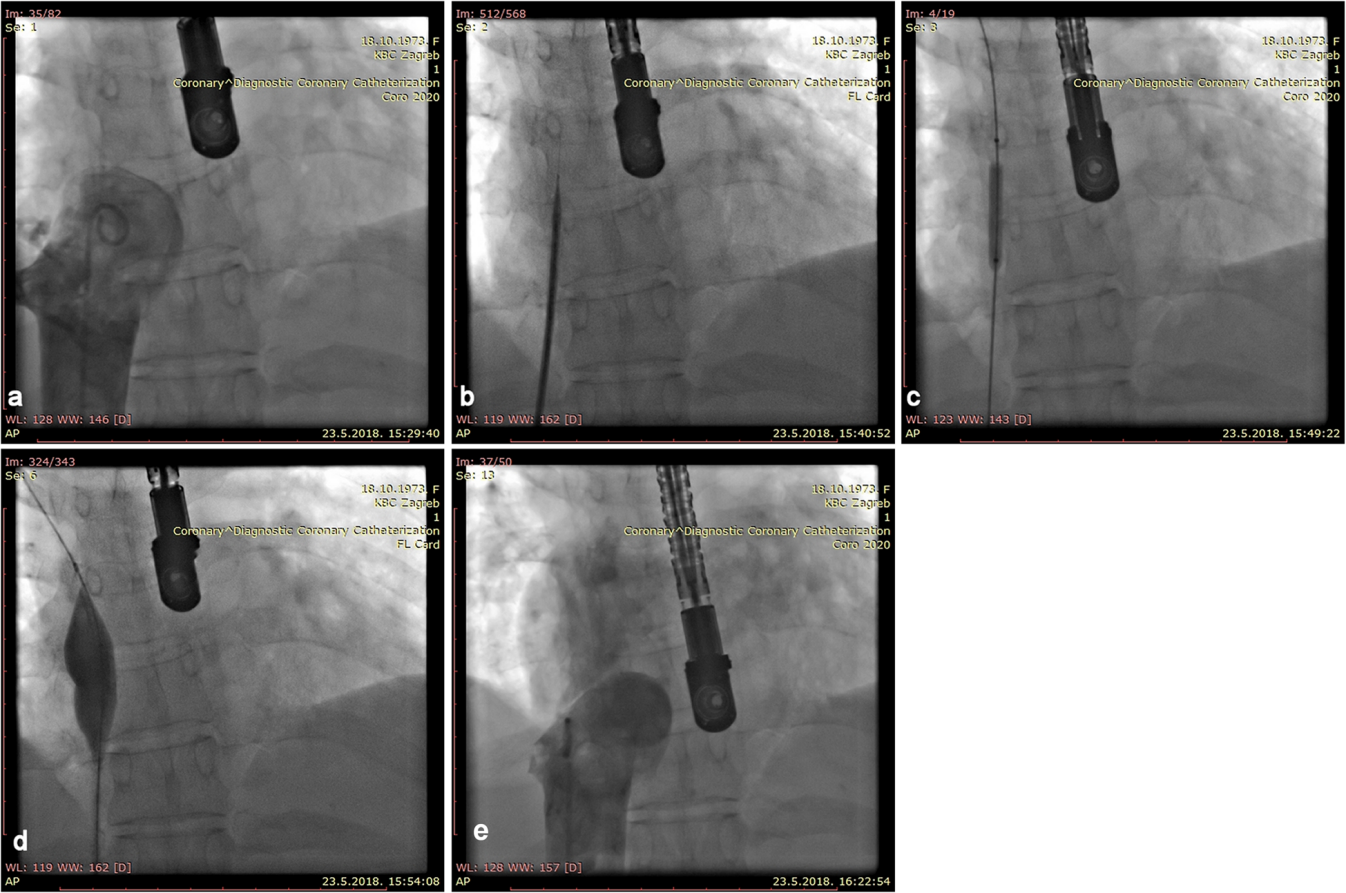
INTERVENTION **a** Angiography shows a complete obstruction of the blood flow between IVC and RA, **b** puncture with a Brockenbrough needle, **c** dilatation of the membrane with a 6/20 mm NC balloon. **d** dilatation with a 16/20 mm balloon. **e** final angiography of IVS, after withdrawn of a 30/40 balloon through the membrane, showing restoration of IVC to RA flow patency

Percutaneous intervention was indicated. The patient was primed with 300 mg of Clopidogrel and 325 mg of Aspirin. Venous access was obtained via 6 French sheaths in the right femoral vein. Unfractionated heparin (5.000 units) was given. Venography was performed via pigtail catheter. It revealed a short, completely occluded segment (thick web) between the right atrium (RA) and IVC. After an unsuccessful attempt to puncture the membrane with a distal tip of an Amplatz stiff wire (J curve, 0,035 Cook Medical, Bloomington, IN, USA), the thick web was punctured with a curved needle through a transseptal introducer sheath (Mullins Sheath 8 Fr and Brokenborough needle 18 gauge, Medtronic, Minneapolis, MN, USA), in the same manner as we would puncture the interatrial septum. After confirming the position of the needle by contrast injection, the sheath was advanced over the needle into the RA, beyond the occlusion. Then a 0.032 J-tip wire was negotiated across the web and was parked in the right subclavian vein. The membrane was first dilated with an available 6,0/20 mm non compliant coronary balloon (NC Emerge, Boston Scientific, Boston MA, USA), with 16 atm, followed by dilatation with a bigger non-complined peripheral balloon16/20 mm with 20 atm (Atlas Gold, Bard Vascular, Tempe AZ USA), with poor result. Sequentially, several balloon dilatations with 30/40 mm semi-compliant balloon (Z-med II, Numed-Braun, Bethlehem, PA, USA), with 4 atm pressure was performed, resulting in immediate recoil. Satisfactory result was achieved after the inflated big balloon was sharply withdrawn through the membrane (a “Rashkind-like” manoeuvre). Stenting was not possible, due to the membrane position (Fig. 3).

**Fig. 4:**
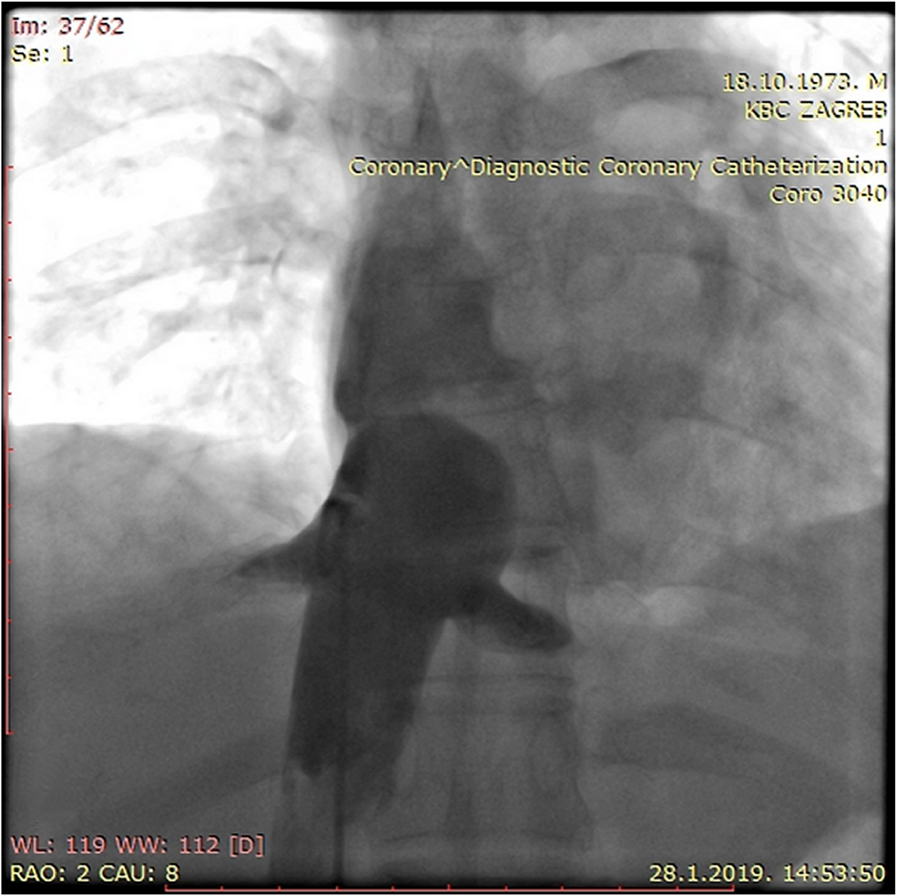
CAVOGRAPHY: venography after 3 months shows the sustained result of the previous intervention

There was no procedural or late complication. The patient recovered very well, and a permanent anticoagulation therapy with warfarin was introduced. The first follow-up was done three months afterwards. Clinically, the patient felt well, there was no evidence of ascites or a peripheral edema. A reduction of collaterals, and only a discrete hepatomegaly was found. An invasive venography was performed, with a planned repeated intervention, if necessary. The result was considered good, with even improved communication throughout the membrane, in comparison with the post-intervention result, so the re-intervention was not performed (Fig. 4). A permanent anticoagulation therapy was recommended to the patient and at the 12-month follow up, she was without any problems.

## Discussion

BCS is defined as a hepatic venous outflow track obstruction at any level: from small hepatic veins to right atrium-IVC junction, regardless of the cause of obstruction (Janssen et al. 2003). Membrane-like obstruction commonly found in angiography, consist of organized old thrombus, arranged in layers of different ages. The clinical onset is mild and causes liver damage by congestion, as was the case in our patient. A distinct pattern of subcutaneous venous collaterals typically develops together with retroperitoneal collaterals through the ascending lumbar and the iliolumbar veins into the hemiazygos and azygos veins.

The previous congenital vascular malformation theory no longer holds, because the disease occurs mostly in adulthood, and the transformation of thrombosis into a membrane has now been well-documented, both, pathologically and clinically. Regardless, our patient was an adult congenital heart disease patient, with a history of pulmonary embolism and possible deep venous thrombosis. We found no evidence of thrombophilia.

The best imaging methods for establishing the diagnosis, are the ultrasound, CT and MRI. Once the diagnosis is established, IVC venography is needed to guide the endovascular techniques. In our patient, the ECHO was first misleading, and MRI was the key diagnostic tool. In BCS with membranous obstruction of IVC, balloon dilatation and stenting should be the treatment of choice with an expected good long-term result (Darwish Murad et al. 2009). The intervention was chosen as the best therapeutic option in our patient’s case. In the absence of any communication through the membrane, a technique similar to trans-septal puncture was applied (Yang et al. 1996).

Different balloons were used in literature. The use of Inoue balloon was popularized, for its clear advantages and was used in many cases (Poddar et al. 2014). We used a Z-Med II balloon (Numed/Braun), regularly available in our cath-lab, with a good result. With a standard ballooning technique, however, we did not achieve a lasting result, because of the recoil after repeated balloon inflations. The improved flow was reached after the thick membrane was pulled apart with the big balloon.

## Conclusion

Balloon angioplasty is effective in relieving IVC obstruction with minimal morbidity. Stents should be used whenever there is a residual obstruction or restenosis after balloon angioplasty. Follow-up results are favourable in both balloon angioplasty and/or stenting. We present a case of interventional balloon disruption of a membrane obstructing IVC outflow to the RA, in a patient uneligible for stenting. A good result was achieved by balloon angioplasty alone, despite the recoil, with an unusual technique.

## Data Availability

All data generated or analyzed during this study are included in this published article.
